# An App-Based Physical Activity Intervention in Community-Dwelling Chinese-, Tagalog-, and Vietnamese-Speaking Americans: Single-Arm Intervention Study

**DOI:** 10.2196/56373

**Published:** 2024-06-10

**Authors:** Antony Nguyen, Filmer Yu, Linda G Park, Yoshimi Fukuoka, Ching Wong, Ginny Gildengorin, Tung T Nguyen, Janice Y Tsoh, Jane Jih

**Affiliations:** 1 Division of General Internal Medicine University of California San Francisco San Francisco, CA United States; 2 Department of Epidemiology Columbia University Mailman School of Public Health New York, NY United States; 3 Asian American Research Center on Health San Francisco, CA United States; 4 Department of Community Health Systems University of California San Francisco San Francisco, CA United States; 5 Department of Physiological Nursing University of California San Francisco San Francisco, CA United States; 6 Multiethnic Health Equity Research Center University of California San Francisco San Francsico, CA United States; 7 Department of Psychiatry and Behavioral Sciences University of California San Francisco San Francisco, CA United States

**Keywords:** physical activity, mHealth, mobile health, mobile app, Asian Americans, physical activity tracker, mobile phone, app, apps, application, applications, app-based, intervention, interventions, community-dwelling, tracker, trackers, pilot study, feasibility, acceptability, cultural, culturally, linguistic, linguistically, evidence-based, community-based, sociodemographic, lifestyle, Chinese, Vietnamese, Filipino, adult, adults, multicomponent, multilingual

## Abstract

**Background:**

Physical inactivity is associated with adverse health outcomes among Asian Americans, who exhibit the least adherence to physical activity guidelines compared with other racial and ethnic groups. Mobile app–based interventions are a promising approach to promote healthy behaviors. However, there is a lack of app-based interventions focused on improving physical activity among Asian Americans whose primary language is not English.

**Objective:**

This pilot study aimed to assess the feasibility and acceptability of a 5-week intervention using a culturally and linguistically adapted, evidence-based mobile phone app with an accelerometer program, to promote physical activity among Chinese-, Tagalog-, or Vietnamese-speaking Americans.

**Methods:**

Participants were recruited through collaborations with community-based organizations. The intervention was adapted from a 12-month physical activity randomized controlled trial involving the app and accelerometer for English-speaking adults. Sociodemographic characteristics, lifestyle factors, and physical measurements were collected at the baseline visit. A 7-day run-in period was conducted to screen for the participants who could wear a Fitbit One (Fitbit LLC) accelerometer and complete the app’s daily step diary. During the 4-week intervention period, participants wore the accelerometer and reported their daily steps in the app. Participants also received daily messages to reinforce key contents taught during an in-person educational session, remind them to input steps, and provide tailored feedback. Feasibility measures were the percentage of eligible participants completing the run-in period and the percentage of participants who used the app diary for at least 5 out of 7 days during the intervention period. We conducted poststudy participant interviews to explore overall intervention acceptability.

**Results:**

A total of 19 participants were enrolled at the beginning of the study with a mean age of 47 (SD 13.3; range 29-70) years, and 58% (n=11) of them were female. Of the participants, 26% (n=5) were Chinese, 32% (n=6) were Vietnamese, and 42% (n=8) were Filipino. All participants met the run-in criteria to proceed with the intervention. Adherence to the app diary ranged from 74% (n=14) in week 2 to 95% (n=18) in week 4. The daily average steps per week from accelerometers increased each week from 8451 (SD 3378) steps during the run-in period to 10,930 (SD 4213) steps in week 4. Participants reported positive experiences including an increased motivation to walk and the enjoyment of being able to monitor their physical activity.

**Conclusions:**

This is the first pilot study of a multicomponent intervention and evidence-based mobile phone app to promote physical activity among Asian Americans who use apps in traditional Chinese, Tagalog, or Vietnamese, which demonstrated high feasibility and acceptability. Future work focused on multilingual mobile apps to address disparities in physical inactivity among Asian Americans should be considered.

## Introduction

While it is well known that physical activity can prevent and moderate chronic diseases, physical inactivity remains a major public health issue and is projected to account for US $301.8 billion in public health care costs globally between 2020 and 2030 [1]. Physical inactivity among Asian Americans, 1 of the fastest-growing racial or ethnic populations in the United States, has increased from 21.5% in 2011-2012 to 32.4% in 2015-2016 [2,3]. Asian Americans had the lowest mean physical activity minutes and the percentage of sufficient physical activity, based on the national guidelines, in comparison with African American, Latino or Hispanic American, and non-Hispanic White populations [4,5]. Moreover, adverse social determinants of health, such as acculturation, negatively influence physical activity among Asian American adults and increase the risk for poor health outcomes, such as metabolic syndrome [6-8].

These low levels of physical activity are worrisome since physical inactivity has been associated with poor mental and physical health outcomes, including psychological distress [9], gastrointestinal conditions (eg, nonalcoholic fatty liver disease [10], colon cancer risk [11], and diabetes [12]), and cardiovascular risk factors (eg, hypertension [13], dyslipidemia [14], and obesity [15]). The level of inactivity is even more concerning when the data are disaggregated by Asian ethnic groups. For example, only 17.8% of Chinese Americans and 14.2% of Vietnamese Americans from Philadelphia and New Jersey met the physical activity recommendations [16]. Furthermore, among Asian ethnic groups, Filipino Americans have the highest prevalence of both diabetes and cardiovascular disease in California [17,18]. To help address these health disparities, programs that blend culturally relevant principles with biomedical guidelines have shown success in improving health knowledge and empowerment [19]. More culturally and linguistically relevant interventions to promote physical activity are needed among Asian Americans.

With the ubiquity of smartphone use, mobile phone app–based interventions could help promote and support healthy behavior change [20-23]. However, there has been limited research on the use of apps to improve health behaviors, including physical activity, among Asian Americans, particularly those whose primary language is not English [24]. Therefore, we adapted an efficacious mobile phone app with an accelerometer program [25-27] and evaluated the feasibility and overall acceptability of both the program’s app usage and physical activity tracking among diverse English-speaking populations for Asian Americans that prefer to use the apps in Chinese, Tagalog, or Vietnamese in a pilot study.

## Methods

### Study Design

This was a single-arm, 5-week (1-week run-in period and 4-week intervention period), pilot interventional study aimed to assess the feasibility and acceptability of a cultural and linguistic adaptation of an evidence-based mobile phone app with an accelerometer program to promote physical activity [25,26,28] among Asian Americans who speak Chinese, Tagalog, or Vietnamese.

### Ethical Considerations

Study requirements were explained to participants and written consent was obtained. Participants received up to $50 for completing all study components ($20 at baseline, $10 at post run-in, and $20 at the end of the study). Participants who used their own iPhone (Apple, Inc.) received an additional $30 for mobile service reimbursement. The institutional review board at the University of California San Francisco (UCSF) approved all study procedures (13-10787).

### Cultural and Linguistic Adaptation of the Intervention

The adapted intervention consists of (1) one brief in-person individual education session and (2) a 4-week intervention using a mobile app and Fitbit One. The in-person education session included seven domains: (1) overview of the physical activity program and tailored short and long-term goal setting based on each participant’s baseline physical activity data measured by Fitbit One, (2) education about the duration and intensity of brisk walking and the health benefits of physical activity, (3) discussion of barriers to increase physical activity and development of strategies to overcome these barriers, (4) value and identification of social support for physical activity, (5) relapse prevention, (6) healthy diet and weight maintenance, and (7) safety of the physical activity. The adapted app has two main functions: (1) a daily message or video clip and (2) a daily diary to record total daily steps and type and duration of completed physical activities. The daily messages and video clips reinforced the 7 domains addressed in the brief in-person intervention. The daily physical activity diary appeared between 7 PM and midnight. If no diary entry was made by 8:30 PM, an automated text message was sent to remind the participant to record their total daily steps and the type and duration of physical activities performed. An automated text message was also sent if a participant did not use the app for 3 consecutive days. Weekly step goals, displayed in the weekly goals option, were automatically increased by 20% each week, which was aligned with the original intervention protocol, relative to the participant’s run-in average, until a goal of 10,000 steps per day and 7 days per week was reached [25,26,28].

**Figure 1:**
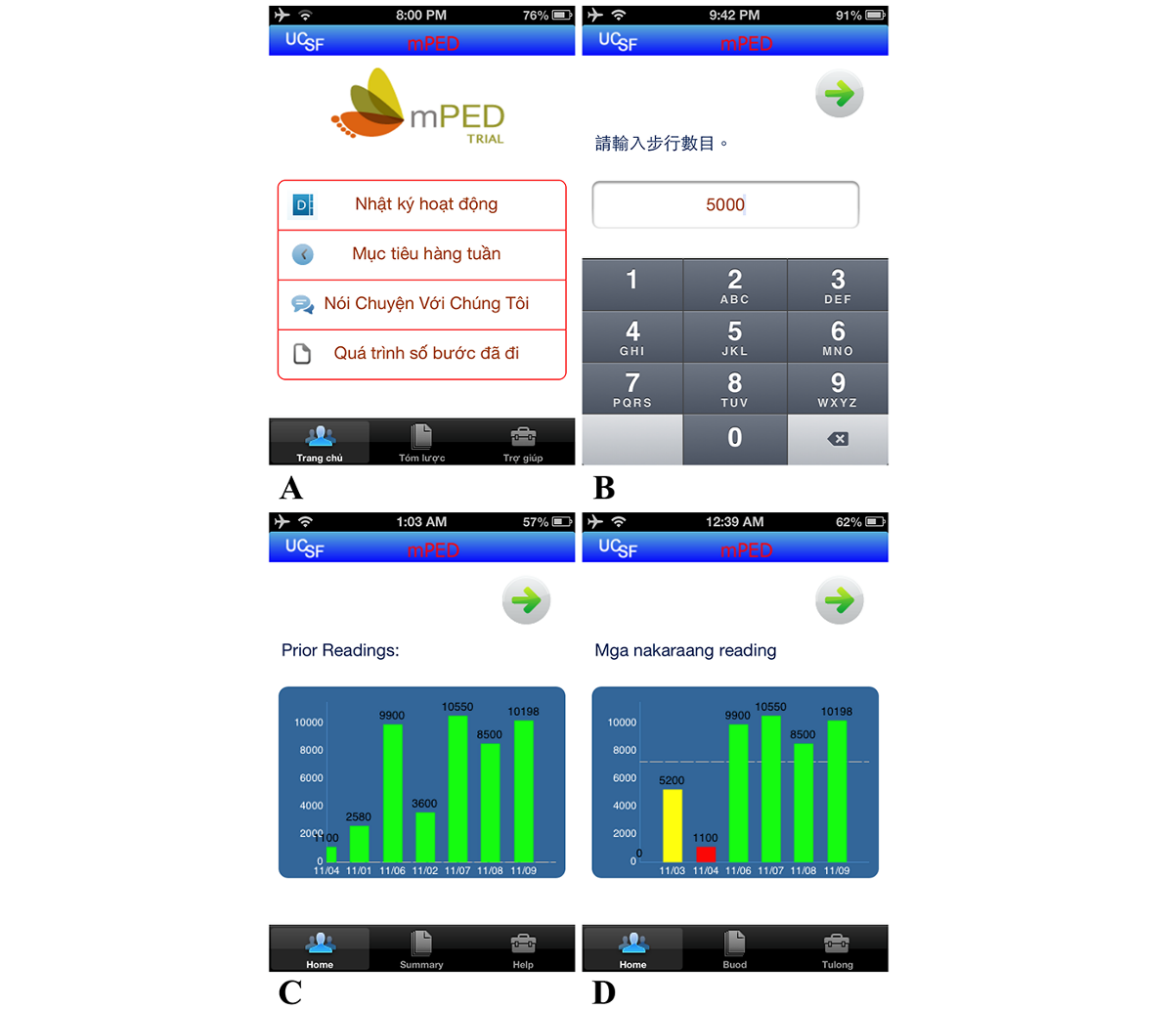
Screenshots of the study mobile app. (A) The Vietnamese version of the trial app. The app was also available in Tagalog and traditional Chinese. Translations: "Nhật ký hoạt động" = Activity diary; "Mục tiêu hàng tuần" = Weekly goals; "Nói Chuyện Với Chúng Tôi" = Talk to Us; "Quá trình số bước đã đi" = Steps History; "Trang chủ" = Home; "Tóm lược" = Summary; "Trợ giúp" = Help. (B) The mobile app daily diary step entry in traditional Chinese (translation: Weekly Steps). (C and D) Mobile app data visualization of steps by day as entered by the participants in English and Tagalog. Translation: "Mga nakaraang" = Prior. UCSF: University of California San Francisco.

With a bicultural and bilingual research team, we adapted the intervention for Chinese, Tagalog (Filipino), and Vietnamese speakers. Using a team approach, all participant-facing intervention materials were forward translated to traditional Chinese, Tagalog, and Vietnamese followed by a critical review of the cultural and language equivalence at a fourth-grade reading level by a team of culturally and linguistically competent staff members and researchers. We conducted pretesting of translated and adapted intervention materials with 4 bicultural and bilingual adult community members, 2 who spoke and read Chinese, 1 who spoke and read Tagalog, and 1 who spoke and read Vietnamese. Intervention materials were further revised by incorporating feedback obtained from pretesting. With the Mobile Services Group of the UCSF Information Services Unit, we developed an iOS (Apple Inc)–based study app, because iOS is the most used mobile phone operating system by Asian Americans [29]. The study app had all the interventional components and features of the parent app. Figure 1 shows screenshots of the app in the 3 Asian languages.

### Participant Recruitment

We recruited a convenience sample in collaboration with 3 San Francisco Bay Area community–based organizations with whom we had existing partnerships—NICOS Chinese Health Coalition, the Bayanihan Community Center, and the Southeast Asian Community Center. Our community partners have long histories of serving the Chinese, Filipino, and Vietnamese communities, respectively. In addition, we focused on these Asian subgroups as they account for more than half of the Asian Americans, experience physical activity–related health disparities [30-34], and are a majority of the East and Southeast Asian community in the San Francisco Bay Area [35]. Community partners distributed multilingual flyers to assist with the recruitment. Our research team also used emailing and telephone recruitment of Chinese and Vietnamese participants of prior research studies who consented to be recontacted for new studies. The study enrolled the first participant in November 2013, and the last participant completed the intervention in July 2014.

### Participant Eligibility

Participant inclusion criteria were (1) self-identification as Chinese, Filipino, or Vietnamese; (2) able to speak and read Chinese (Cantonese and Mandarin for spoken Chinese, and traditional Chinese for written Chinese), Tagalog, or Vietnamese; (3) aged between 25 and 70 years; (4) intended to be physically active; (5) had access to a home or mobile phone; and (6) was current or past mobile phone user. Current access to or possession of an iOS mobile phone was not required because we provided a loaner iOS mobile phone as needed. Exclusion criteria were (1) had known medical conditions that require special attention in an exercise program (eg, prior myocardial infarction, history of angina, and diabetes mellitus with insulin treatment); (2) had known bone or joint problems that impair moderate physical activity; (3) were pregnant or within 6 months post partum; (4) had known severe hearing or visual problems; (5) current participation in lifestyle modification programs; or (6) plans to travel internationally during the study period.

### Study Procedures

At the screening or baseline visit, research staff conducted a baseline assessment, which included a collection of sociodemographic characteristics and lifestyle factors (eg, self-rated health status, cigarette use, and alcohol intake) in a private office at UCSF or a community-based organization site. Anthropometric and blood pressure measures were also assessed. Participants then received hands-on training on using a Fitbit One accelerometer and the mobile app with features of daily messaging and step diary. Participants who did not have an iPhone (Apple Inc) were provided with a study iPhone with full phone, texting, and internet services for the duration of the study. During this screening or baseline visit, participants also provided the name of their physician and their physician’s contact information. We sent notification letters of participation in the study to the physicians after the baseline visit for participants with multiple cardiovascular risk factors or other chronic conditions.

To identify participants who were not able to wear a Fitbit One daily and those who did not complete the daily mobile phone diary, we conducted a 7-day run-in period prior to the 4-week intervention study period. During the run-in period, participants were asked to wear a study-provided Fitbit One for at least 8 hours daily. They reported their daily step counts, type of physical activity, and duration through the app diary. Participants were enrolled into the 4-week main study period if they completed the following during the run-in period: (1) wore the Fitbit One for at least 4 out of 7 days, in which they were not sedentary (defined as at least 8 hours of physical activity as shown on the accelerometer) or (2) recorded their physical activity into the mobile phone diary at least 5 out of 7 days.

Following the run-in period and at the start of the 4-week intervention study period, research staff downloaded participants’ daily steps to a secure study computer. They engaged in a brief in-person educational session with a language-concordant team member that lasted up to 30-45 minutes.

During the intervention study period, the study app delivered language-adapted daily messages in the form of yes or no questions and multiple-choice questions to the participants to reinforce key content taught during the educational session. The research staff was able to monitor the responses on a secure internet server. The app also provided an internet-based phone diary to record daily physical activity with instant reinforcing feedback. In-language text messages delivered feedback that was linguistically and culturally tailored from the original intervention protocol to each participant’s specific activity, number of steps, and daily completion status. Participants received automatic reminders via text messages in their preferred language from Salesforce (Salesforce Inc) using Twilio (Twilio Inc)—one to remind them to log in to their app to read the daily message at noon if the participant did not log in and another text message in the evening to record their daily steps into the phone diary. A participant’s individualized physical activity goals were automatically updated with a 20% step increase per week based on their baseline step counts as per the original intervention protocol [27,36].

Research staff downloaded participants’ daily step diary entries to a secure study computer at the end of the interventional study period. Participants received a US $50 incentive for completing all study visits—US $20 at baseline, US $10 in the post–run-in period, and US $20 at the end of the study. The research team used an end-of-study assessment with a survey and open-ended questions to obtain ratings and feedback on their experience and satisfaction with the intervention.

The UCSF Information Services Unit supported the study team in data collection in Salesforce from the study app throughout the entire study. Fitbit One data for up to 30 days were collected from Fitabase, a comprehensive data management platform for Fitbit One devices, and downloaded to an encrypted computer.

### Participant and Physical Activity Measures

Sociodemographic characteristics (eg, sex, age, race or ethnicity, education level, annual household income, and marital status) were collected at baseline. Health-related status and lifestyle factors (eg, self-rated health status, cigarette use, and alcohol intake) were collected at baseline and at the end of the study. The research team also collected internet, mobile phone, and past physical activity use information. We also collected (1) percentage adherence to the daily phone diary and (2) the level of physical activity in terms of the total number of steps per day as measured by the Fitbit One. The number of steps per week of each person was calculated as the average value across the days with at least 8 hours of nonzero step data.

### Feasibility and Acceptability Measures

Feasibility was only examined for recruitment into the study and adherence to the intervention. Feasibility for recruitment was assessed by the percentage of eligible participants completing the 7-day run-in period and enrolled in the study. The feasibility of intervention adherence was evaluated by the percentage of participants who used the mobile app’s diary for at least 5 out of 7 days. The threshold for feasibility was set to be 80% based on initial work with the evidence-based mobile phone app with an accelerator program [25,28]. Acceptability was examined through participant responses to open-ended questions regarding participant feedback, experience, and satisfaction. Participant responses were collected during the postintervention interviews that included questions on user satisfaction and experience: “What did you learn the most from the study?” “What did you like the most about the study?” “What did you like the least about the study?” “What would you change about the study?” “What do you feel will motivate you more to maintain your physical activity?” and “What advice would you give to others to help increase their physical activity?”

### Statistical Analyses

We computed frequency counts and summary statistics for participants’ characteristics at baseline. Data from the Fitbit One accelerometer were retrieved using Fitabase. Daily steps were computed when there were data (nonzero step) for at least 8 hours over a 24-hour period. The computed daily steps were then used in the computation of the mean and SD of the daily steps for each week (run-in and weeks 1 to 4). Quantitative analyses were conducted in SAS (SAS Institute).

### Qualitative Analysis

Qualitative data were analyzed using thematic analysis by 2 coders to identify the themes related to the acceptability of the intervention including user satisfaction and experience. Interviewers took detailed notes in English and these qualitative responses were entered and stored in a REDCap (Research Electronic Data Capture; Vanderbilt University) database. Qualitative responses were exported in a Microsoft Excel document for analysis. Both coders initially independently reviewed and coded participant responses to each individual question guided by the postintervention interview guide and acceptability domains of interest that were asked during the postintervention follow-up interviews. Acceptability domains of interest included the perceived impact of and participant learning from the intervention and barriers and proposed changes to the intervention. The coders then met to discuss initial codes, resolve any discrepancies, build consensus, and finalize the codebook. All qualitative participant responses were then recoded by the 2 coders using the finalized codebook.

## Results

### Patients’ Recruitment and Characteristics

We recruited and enrolled a total of 19 participants in this pilot study. Study participants were 26% (n=5) Chinese, 32% (n=6) Vietnamese, and 42% (n=8) Filipino (Table 1). The mean age was 47 (SD 13.26; range 29-70) years, and 58% (n=11) were female. A total of 89% (n=17) used a computer or had access to the internet at least once a week during the last month, and 95% (n=18) used a mobile phone at least once a week in the past months prior to the study enrollment. However, 63% (n=12) of the participants were provided an iPhone for the study and 79% (n=15) never used an activity tracker to monitor steps before the study. Nearly all participants (n=18, 95%) reported during the baseline visit having prior experience of using a computer, internet, and mobile phone.

All participants met the 7-day run-in criteria by wearing a Fitbit One accelerometer for at least 8 hours a day for at least 4 days or entering their daily steps on at least 5 days during the 7-day run-in period. During the run-in period, on average, participants wore their Fitbit One for 5.42 (SD 1.98) days. All but 1 participant met the criterion to wear the Fitbit One for at least 8 hours a day for at least 4 days to provide daily step data. The 1 participant who did not meet the Fitbit One criterion did, however, enter the number of steps using the app diary for 6 days during the run-in period. For the number of days using the app diary to enter daily steps (at least 5 out of 7 days), all but 2 individuals met this criterion. Both of these participants, however, were able to provide Fitbit One steps data on all 7 days, wearing the accelerometer for at least 8 hours daily. Of note, the most common reason for missing the entry of steps in the diary was because they passed the midnight cut-off time for entering the number of steps of the day. All participants proceeded to the intervention phase and attended an in-language educational session with a language-concordant team member. A total of 18 (95%) out of 19 participants remained until the end of the study.

**Figure 2:**
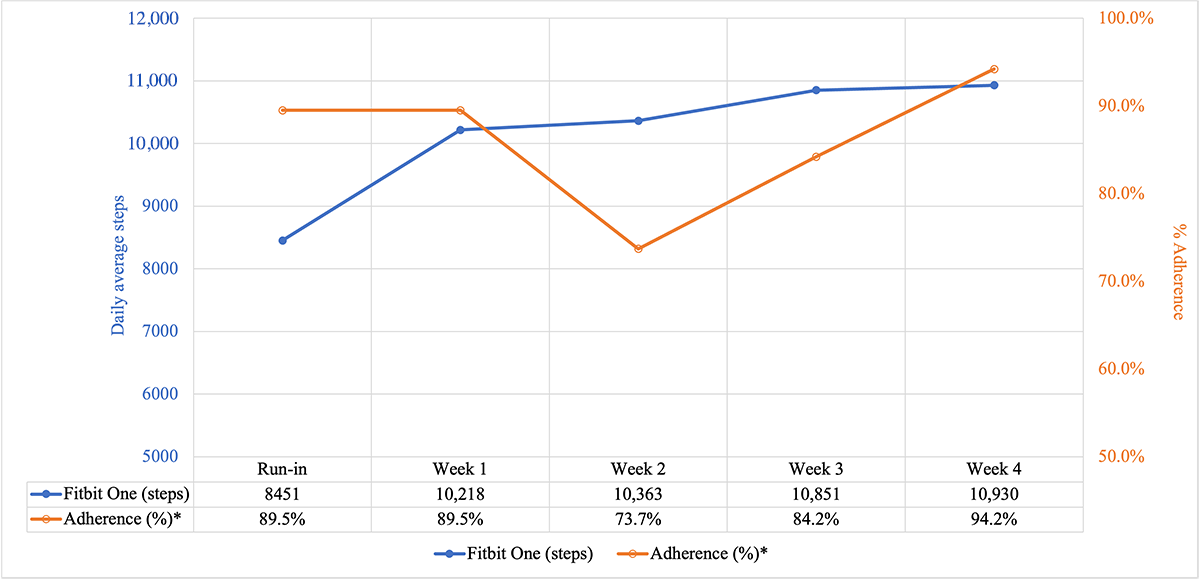
Recorded daily average steps by Fitbit One accelerometer and percentage of app diary adherence* by week. *Adherence was defined as the percentage of participants who used the app diary on at least 5 days out of 7 days.

Figure 2 in orange shows the percentage of patients adhering to entering daily steps into the app diary for at least 5 days out of the 7 consecutive days in a week during the run-in period and for the 4-week intervention period. Regarding app diary usage feasibility, adherence ranged from 74% (14/19) in week 2 to 95% (18/19) in week 4.

**Table 1:** Baseline characteristics of study participants (N=19).

Characteristics				Values
**Sociodemographic characteristics**				
	**Asian ethnicity, n (%)**			
		Chinese	5 (26)	
		Vietnamese	6 (32)	
		Filipino	8 (42)	
	**Age (years)**			
		Mean (SD)	47.3 (13.3)	
		Range	29-70	
	Female, n (%)		11 (58)	
	**Education, n (%)**			
		High school diploma or less	3 (16)	
		Some college	4 (21)	
		Completed college abroad	12 (63)	
	**Annual household income (US $), n (%)**			
		<$20,000	2 (11)	
		$20,000-$40,000	8 (42)	
		>$40,000	5 (26)	
		Do not know or decline to state	4 (21)	
	**Marital status, n (%)**			
		Never married	7 (37)	
		Currently married	10 (53)	
		Divorced or widowed	2 (11)	
**Health-related status and lifestyle factors**				
	**BMI^a^ (kg/m^2^), n (%)**			
		<18.5 (underweight)	1 (5)	
		18.5-22.9 (healthy weight)	8 (42)	
		23-26.9 (overweight)	6 (32)	
		>27 (obese)	3 (16)	
		Missing	1 (5)	
	**Self-rated health status, n (%)**			
		Fair or poor	1 (5)	
		Good	13 (68)	
		Very good or excellent	5 (26)	
	Reported having high blood pressure or taking a blood pressure medication, n (%)		4 (21)	
	**Reported having total cholesterol >200 mg/dL, n (%)**			
		No	11 (58)	
		Yes	4 (21)	
		Do not know	4 (21)	
	**Reported having HDL^b^<40 mg/dL, n (%)**			
		No	7 (37)	
		Yes	3 (17)	
		Do not know	9 (47)	
	**Reported cigarette use in the past week, n (%)**			
		No	17 (90)	
		Yes	1 (5)	
		Missing	1 (5)	
	**Number of alcoholic drinks per week during the past month, n (%)**			
		None	12 (63)	
		<1	3 (16)	
		1-2	2 (11)	
		3-7	2 (11)	

^a^BMI cutoffs are based on the World Health Organization–recommended cutoffs for Asian and Asian American populations [15,30,37,38].

^b^HDL: high-density lipoprotein.

Figure 2 also shows the average number of daily steps by week in blue as measured by the Fitbit One accelerometer during the 7-day run-in period and 4-week intervention period. The recorded daily average steps per week from participants’ accelerometers increased each week. For the 7-day run-in period, the recorded daily average steps among participants were 8451 (SD 3378) steps. For week 1 of the 4-week study period, the recorded daily average steps were 10,218 (SD 9343) steps, and for week 2, the recorded daily average steps were 10,364 (SD 4530) steps. The recorded daily average steps increased to 10,851 (SD 4528) steps for week 3 and continued to increase to 10,930 (SD 4213) steps for week 4. During the 4-week study period, the mean number of days per week with daily steps data from the accelerometer ranged from 5.58 (SD 1.87) to 6.10 (SD 1.76) days. Regarding the percentage of participants adherent to wearing the accelerometer for at least 8 hours a day for at least 4 days a week during the intervention period, in all weeks except week 2, adherence exceeded the 80% feasibility threshold, with 90% (17/19) of participants adhering in week 1, a total of 74% (14/19) in week 2, a total of 84% (16/19) in week 3, and 95% (18/19) in week 4.

### Qualitative Results

Overall, the participants expressed high acceptability for this intervention. The participants reported increasing motivation to walk more and liked being able to monitor their activity while being active. In 1 instance, a participant said, “I wanted to improve my steps when it was lower than my ideal goal.” Participants also enjoyed using the activity tracker and having an accompanying mobile app to help track and monitor their progress and goals. A participant said, “I liked comparing my steps each day and challenging myself to walk more or work out.”

Although the overall feedback was positive, the participants also provided constructive comments and suggestions regarding responding to prompts, reminders, and improvement of the mobile app. For example, 1 participant mentioned “I would like to know the calories I burned each day.” Despite the intervention being focused on the individual participant, many felt that “having a gym/work out buddy” or “a group of people highly motivated to accomplish goals” would help maintain their physical activity. However, most participants also cited overall health as an effective incentive to be physically active, with 1 participant saying he would “have more energy to play with baby.”

## Discussion

### Principal Findings

To our knowledge, this is the first pilot interventional study focused on a culturally and linguistically adapted, multicomponent intervention, and evidence-based mobile phone app to promote physical activity among Chinese-, Tagalog-, or Vietnamese-speaking Americans. This pilot study showed high feasibility of the app-based physical activity intervention with all participants who enrolled in the study meeting run-in period criteria and all except 1 participant remaining until the end of the study. The percentage of participant adherence to the usage of the mobile app diary component each week was high meeting the feasibility threshold except in week 2 of the intervention period with adherence at 74% (14/19). The variation in week-to-week adherence may be attributed to the participants forgetting to enter their daily steps before the midnight deadline.

Participants’ feedback in postintervention interviews suggested a strong overall acceptability of this in-language multicomponent intervention including an evidence-based mobile phone app for promoting physical activity. Participants noted how the in-language intervention provided a means to monitor their physical activity and motivated them to set goals. Additionally, our study had a promising trend of weekly increases in average daily steps recorded by the participants from the run-in period to the end of the 4-week intervention study period.

### Comparison With Prior Works

Our pilot study findings are the first to our knowledge to deliver a multicomponent intervention including a mobile app in multiple Asian languages to promote physical activity among Asian Americans. Our study adds to 1 published pilot study that used a multicomponent, culturally adapted intervention including an accelerometer and a mobile app with a diary in English to promote physical activity and healthy eating for weight loss among adult Filipino Americans with type 2 diabetes mellitus that showed the feasibility and potential efficacy [39]. However, other prior studies related to mobile health (mHealth) technologies for physical activity promotion among Asian adult populations have focused on populations outside of the United States [40-42] and pediatric and young adult populations [43-47], such as adolescents and university students. In addition, none of these studies included the use of an accelerometer and mobile-based app in conjunction to one another [40-47].

Furthermore, participants in poststudy interviews hinted at the potentially essential role of social networks and support to promote physical activity which are important factors that influence adherence to mHealth apps and interventions [48]. For instance, integration of customizable options, adding social features (eg, social comparisons and challenges), and incorporating communication with health care clinicians—all of which were not features of this intervention—may be beneficial to increase adherence to the intervention components. Social features that underscore social support, in particular, may be essential in promoting physical activity as previous literature has reported a positive association between social support and physical activity in older adults [49] and an increase in physical activity knowledge and self-reported physical activity among older Chinese and Vietnamese Americans in health promotion interventions involving in-language physical activity education and social support [31,32].

### Limitations

Several limitations should be considered in the context of the study findings. First, this is a pilot with a modest sample size from 1 geographic location. Further, as this was single-arm pilot feasibility and acceptability study without a comparison group for a relatively short study period, while there was a trend of the increased number of daily steps during the study period, the efficacy of the intervention to promote maintained, increased physical activity could not be ascertained. We evaluated the overall feasibility and acceptability of the adapted intervention, not individual intervention components. In addition, given the mean age and level of educational attainment of the study sample, the generalizability of the study results may not be applicable to younger and older adults and individuals of lower educational attainment. It is also noteworthy that the majority of our sample completed a college degree or beyond and opted to receive an iPhone for the study. Future studies should examine the feasibility and acceptability of such interventions across samples with varying educational attainment and capture technology literacy to observe trends in technology use between different levels of education. Participants may have opted to use a study-provided iPhone to not have to use their own smartphone devices or have owned an Android (Google) device. Interventions involving apps should seek to develop mobile apps in both iOS and Android operating systems.

Moreover, this study was conducted between 2013 and 2014. Since this study, mobile apps have commonly integrated accelerometers, which suggests that similar interventions in the future would be more feasible. However, in-language physical activitypromoting apps in Chinese, Tagalog, or Vietnamese continue to be undeveloped. Future studies should include and examine the usage of such interventions in additional Asian subgroups and languages, especially given the prevalence of obesity and physical activityrelated chronic diseases [50]. This study’s strengths include a multicomponent intervention including an app available in traditional Chinese, Tagalog, and Vietnamese; having study participants who speak and read Chinese, Tagalog, or Vietnamese who are not often included in studies; and engagement with Asian American community–based organizations to recruit participants.

### Conclusions

In conclusion, our study suggests that the adapted evidence-based, mobile appbased intervention to promote physical activity was feasible with strong acceptability among Asian Americans who speak and read Chinese, Tagalog, and Vietnamese. Further refinements of the intervention to include components promoting social support to facilitate increased physical activity could be promising. Future work to address disparities in physical activity among Asian Americans, particularly those who are bilingual or have limited English proficiency, should include consideration of in-language mobile app–based apps.

## Abbreviations

mHealth: mobile health
REDCap: Research Electronic Data Capture
UCSF: University of California San Francisco
